# Determinants and Characteristics of Insulin Dose Requirements in Children and Adolescents with New-Onset Type 1 Diabetes: Insights from the INSENODIAB Study

**DOI:** 10.1155/2023/5568663

**Published:** 2023-11-26

**Authors:** Maude Beckers, Olivier Polle, Paola Gallo, Noémie Bernard, Céline Bugli, Philippe A. Lysy

**Affiliations:** ^1^Paediatric Endocrinology and Diabetes Unit, Specialized Pediatrics Service, Cliniques universitaires Saint-Luc, Belgium; ^2^PEDI Laboratory, Institut de Recherche Expérimentale et Clinique, UCLouvain, Brussels, Belgium; ^3^Louvain School of Statistics, Biostatistics and Actuarial Sciences, UCLouvain, Louvain-la-Neuve, Belgium

## Abstract

**Aims:**

New-onset type 1 diabetes mellitus (T1D) in pediatric patients represents a clinical challenge for initial total daily insulin dosing (TIDD) due to substantial heterogeneity in practice and lack of consensus on the optimal starting dose. Our INSENODIAB (INsulin SEnsitivity in New Onset type 1 DIABetes) study is aimed at (1) exploring the influence of patient-specific characteristics on insulin requirements in pediatric patients with new-onset T1D; (2) constructing a predictive model for the recommended TIDD tailored to individual patient profiles; and (3) assessing potential associations between TIDD and patient outcomes at follow-up intervals of 3 and 12 months.

**Methods:**

We conducted a comprehensive analysis of medical records for children aged 6 months to 18 years, hospitalized for new-onset T1D from 2013 to 2022. The study initially involved multivariable regression analysis on a retrospective cohort (rINSENODIAB), incorporating baseline variables. Subsequently, we validated the model robustness on a prospective cohort (pINSENODIAB) with a significance threshold of 5%. The model accuracy was assessed by Pearson's correlation.

**Results:**

Our study encompassed 103 patients in the retrospective cohort and 80 in the prospective cohort, with median TIDD at diagnosis of 1.1 IU/kg BW/day (IQR 0.5). The predictive model for optimal TIDD was established using baseline characteristics, resulting in the following formula: TIDD (IU/d) = ([0.09 × Age2] + [0.68 × %Weight Loss] + [28.60 × Veinous pH] − [1.03 × Veinous bicarbonates] + [0.81 × Weight] − 194.63). Validation of the model using the pINSENODIAB cohort demonstrated a significant Pearson correlation coefficient of 0.74. Notably, no significant correlation was observed between TIDD at diagnosis and partial remission markers (IDAA1C, C-peptide) at 3- and 12-months postdiagnosis time points.

**Conclusions:**

In the context of new-onset T1D in pediatric patients, we identified key influencing factors for determining optimal TIDD, including age, percentage of weight loss, weight, veinous pH, and bicarbonates. These findings have paved the way for the development of a dosing algorithm to potentially expedite glycemic control stabilization and facilitate a more individualized approach to treatment regimens.

## 1. Introduction

Type 1 diabetes mellitus (T1D) is a complex metabolic disorder characterized by a progressive destruction of pancreatic *β* cells resulting in insulin secretion deficiency.

T1D represents a major public health issue due to its continuously increasing incidence (i.e., 2-5% yearly from 2002 to 2015) [1–3] and challenges of disease management, which are fueled by a cumulative risk of acute and chronic complications [2].

While insulin therapy and glucose monitoring have been substantially improved by technological developments [4], many unknowns remain about the optimal management of T1D when it comes to initial insulin treatment regimen, preferred insulin delivery device, or location and duration of initial diabetes education. These unknowns create an important heterogeneity in clinical practice between and within countries [5], especially concerning recommendations for insulin dose regimens for children with newly diagnosed T1D. Indeed, the International Society for Pediatric and Adolescent Diabetes (ISPAD) recommends that the insulin starting dose for prepubertal children varies from 0.7 to 1.0 IU/kg body weight (BW)/day [6]. The American Diabetes Association (ADA) suggests an initial total insulin daily dose (TIDD) for children and adolescents ranges between 0.5 and 1.0 IU/kg BW/day while the American Association of Clinical Endocrinologists (AACE) and American College of Endocrinology (ACE) recommend initial insulin doses of 0.4-0.5 IU/kg BW/day [7–9]. Lack of precision of the starting insulin dose may have numerous consequences—all being associated with a delay in achieving metabolic control. For example, duration of hospitalization may be prolonged, or parental fear of glycemic variability (in particular hypoglycemia) might be increased [10]. These particularities influence disease control in the long run, as shown by Samuelsson et al. who emphasized the importance of identifying key factors for early T1D stabilization, as these were correlated both with metabolic control in early adulthood and with the risk of ensuing chronic complications [11].

TIDD is a cardinal parameter for the management of T1D, since it relates to metabolic control and potential side effects of insulinemia [12]. Insulin dosing is complex and dependent on numerous patient characteristics. The following determinants of TIDD at diagnosis were identified: age [13, 14], gender [12, 14], pubertal stage, degree of adiposity [14], vitamin D status [15], thyroid function [16], glycemic control [14], and the occurrence and severity of diabetes ketoacidosis (DKA). At present, it is still unknown if those insulin requirements reflect the acute metabolic disorder at diagnosis or a more specific phenotype with singular outcomes. However, early identification of patients with unusual TIDD levels is key in improving diabetes management and individual disease trajectory [12]. This is to keep in mind, as diabetes control in young children is particularly challenging with significant impact on quality of life and potential long-term diabetes-related complications [10]. Interestingly, some of those clinical and metabolic factors at diagnosis such as age, DKA, or glycemic control are also found to influence the frequency and duration of the partial remission period (PR) [17]. Furthermore, it is well established that the initiation of exogenous insulin treatment in patients with newly diagnosed T1D affords the *β* cell a period of rest, leading to the reversal of glucotoxicity. Consequently, this process facilitates the recovery of both insulin synthesis and secretion [18].

The main goals of our INSENODIAB (insulin sensitivity in new onset type 1 diabetes) study were to explore the influence of patient-specific characteristics on insulin requirements in pediatric patients with new-onset T1D and to construct a predictive model for the recommended TIDD tailored to individual patient profiles. Secondarily, we aimed to assess potential associations between TIDD and patient outcomes at follow-up intervals of 3 and 12 months.

## 2. Materials and Methods

### 2.1. Study Cohorts and Design

INSENODIAB is an observational study conducted at Cliniques universitaires Saint-Luc (CUSL)–UCLouvain, a pediatric endocrinology referral center in Belgium. Patients are treated according to the ISPAD guidelines [1]. On admission, the TIDD is calculated based on the patient body weight and corrected depending on blood glucose levels. When normal blood glucose concentration (70-180 mg/dL [19]) is achieved and diabetes education is completed, the patient is discharged.

For this study, we analyzed the following two distinct cohorts of patients:

1. A retrospective cohort (rINSENODIAB) to investigate determinants and predictive models of TIDD variability during the initial hospitalization
2. A prospective cohort (pINSENODIAB) to validate the predictive model and evaluate potential correlations of initial TIDD with patients' outcomes 3 and 12 months after diabetes onset

rINSENODIAB included children aged 6 months to 18 years old and admitted at CUSL between January 2013 and February 2020 for a new-onset T1D, as per ISPAD guidelines [1]. Data were collected retrospectively through electronic chart review (Epic, Hyperspace). Admissions were identified through the CUSL pediatric diabetology convention registry, and informed consent was obtained from parents.

Patients with the following subsequent characteristics were excluded: (1) use of treatments interfering with insulin secretion and sensitivity (e.g., sulfonylureas, diazoxide, somatostatin analogues, methylxanthine derivatives, corticosteroids, biguanide, and incretins); (2) active inflammatory disease or malignancy at inclusion; (3) hepatic, renal, or adrenal insufficiency; (4) history of bone marrow transplantation; (5) absence of anti-islet autoantibodies (i.e., anti-insulin, anti-IA2, anti-GAD65, and anti-ZnT8); (6) dysmorphia with suspected underlying genetic syndrome; (7) participation in another study within the last 3 months, with the administration of blood derivatives or potentially immunomodulating treatments.

Finally, for the assessment of patient characteristics influencing the insulin dose requirements as well as establishing the predictive models of the recommended TIDD, we excluded patients with mean daily glycemia at discharge out of the 70-180 mg/dL range, as they could not reach an euglycemic state [19].

For all patients, the following historical data were collected:

1. *Clinical Parameters at Diagnosis.* Sex, age, body weight (kg), height (cm), body mass index (BMI, kg BW/m^2^), duration of diabetes symptoms before diagnosis (days, weeks, and months), other medical treatments, and degree of weight loss at diagnosis (in % of initial weight). The percentage of weight loss was either collected through the patient's history or calculated based on the weight at the first medical appointment after discharge, two weeks after initial hospitalization. *Z*-scores (SD) for height, weight, and BMI were adjusted for age and gender according to Belgian reference standards [20]. BMI score evaluation was based on the international BMI cutoffs (International Obesity Task Force) for thinness, overweight, and obesity for children and adolescents [21–23].
2. *Paraclinical Parameters at Diagnosis.* Glycemia (mg/dL), HbA1c (%), presence of DKA defined by veinous pH < 7.3 and/or bicarbonates <15 mmol/L [24], serum creatinine (mg/dL), serum anti-islet autoantibodies (GAD-65 (U/mL), and anti-IA2 (U/mL)).
3. *Paraclinical Parameters during the Initial Hospitalization.* Daily capillary blood glucose levels (mg/dL) at key time points of the day and mean daily glycemia (mg/dL).
4. *Insulin Therapy Regimen during Initial Hospitalization.* Intravenous (IV) or subcutaneous (SC) TIDD were registered daily and expressed in IU/day, IU/kg BW/day adjusted to the weight measured at admission. IV regimen was time-limited and restricted to patients presenting with DKA at onset, as per ISPAD guidelines [6]. The TIDD was calculated by including basal and prandial insulin doses for patients treated by multiple daily insulin injection (MDI) therapy or under continuous SC insulin infusion (CSII). To analyze reliable TIDD, days without 24-hour data completeness were excluded.

To investigate TIDD variability during the hospitalization, we computed a delta TIDD between the first and last day of admission (i.e., TIDD_d_-TIDD_a_) with TIDD_d_ being TIDD at discharge and TIDD_a_ being TIDD at admission. We also created subgroups according to baseline characteristics at diagnosis, that is age (<5 years, 5-10 years, and >10 years), BMI SD (<-2 SD, -2 SD- +1.6 SD, and > +1.6 SD), symptoms duration (<2 weeks, 2-4 weeks, 1-2 months, and >2 months), presence or absence of weight loss, and presence or absence of DKA subgroups. We also defined three insulin sensitivity subgroups based on the TIDD_d_ (IU/kg BW/day) and according to ISPAD recommended starting doses [21]: (1) highly insulin-sensitive patients (HIS) with an insulin requirement below 0.7 IU/kg BW/day, (2) normosensitive patients (NIS) with an insulin requirement in 0.7 to 1.0 IU/kg BW/day range, and (3) insulin-resistant patients (IR) with an insulin requirement above 1.0 IU/kg BW/day.

pINSENODIAB included patients from the multicentric DIATAG cohort (NCT04007809), which was previously described [25] and approved by all participating ethical committees (Comité d'Ethique Hospitalo-Facultaire of CUSL, 2018/04DEC/462). Briefly, pediatric new-onset T1D patients, aged 1 year to 18 years were recruited at diagnosis based on ISPAD criteria [1].

The same clinical and paraclinical parameters as for the rINSENODIAB cohort were collected on admission, adding pubertal status (evaluated by the Tanner staging or dosage of luteinizing hormone levels, LH > 0.3 U/L being considered as entry in puberty) and residual *β*-cell secretion estimates, that is, estimated C-peptide level (CPEP_EST_) [26], insulin dose-adjusted A_1c_ (IDAA_1c_) [27–29], and HbA_1c_ at diagnosis and at 3 and 12 months after diabetes onset. As defined and validated in multiple cohort studies [17, 29, 30], patients with an IDAA_1c_ of 9 or below were considered in PR (remitters).

Insulin therapy regimen at discharge (IU/kg BW/day and IU/day), at 3 and 12 months after diagnosis, was also collected. As in the rINSENODIAB cohort, patients were divided into three insulin sensitivity subgroups.

### 2.2. Statistical Analysis

All statistical analyses were performed using PRISM 9.0, JMP PRO 16.0.0, or R version 4.1.0. To characterize the cohorts, descriptive statistics for discrete (frequencies and percentages) and continuous (medians and ranges or interquartile range) variables were computed for demographics, clinical characteristics, and paraclinical measurements.

To determine the best predictive model of the TIDD (IU/day and IU/kg BW/day), we chose TIDD_d_ because it better reflects the patient's basic needs for a controlled glycemia. All baseline variables from the first day of admission were considered as dependent variables in a multivariable linear regression model applying a backward stepwise approach with a nominal type I error of 5%. Log-normal transformation of laboratory parameters was applied. Adjusted *R*-square and Pearson's correlation between the actual and expected TIDD were used to assess the goodness of fit. The model was subsequently applied on the independent multicentric prospective cohort to evaluate the robustness of the model.

### 2.3. Ethics Approval

An independent, central ethics committee, the Ethics Committee (EC) of Cliniques universitaires Saint-Luc–UCLouvain, approved the study protocol (EC study number: 2020/21JAN/048). The present study was performed in accordance with the Declaration of Helsinki.

## 3. Results

### 3.1. Cohort Description

**Figure 1:**
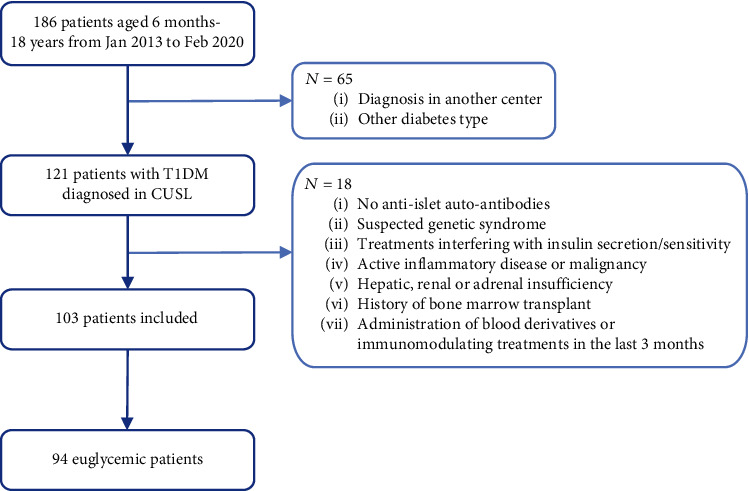
Consort flow of the rINSENODIAB cohort.

One hundred and three children out of 186 admitted with a new-onset T1D were included in the rINSENODIAB cohort (see consort flow in Figure 1), 43.7% of them being males. Median age was 9.2 years old (IQR 6.9), and median weight was 27.0 kg (IQR 20.9). DKA was diagnosed in 37 patients (35.9%), and median HbA_1C_ was 11.5% (IQR 2.6). The median weight loss was 8.7% (IQR 8.6) at admission, and half of patients had been symptomatic for 2 weeks or less. The median TIDD_d_ was 1.1 IU/kg BW/day (IQR 0.5), and most patients were under MDI regimen (92.2%). The median duration of hospitalization was 6.0 days (IQR 5.0).

The pINSENODIAB cohort gathered 80 patients with a new-onset T1D from seven reference centers in Belgium. Median age was 10.6 years (IQR 5.6), median weight was 32.1 kg (IQR 18.7), and 38 patients (47.5%) were pubescent. HbA_1c_ at admission was 12.6% (IQR 2.7) with a median weight loss of 12.8% (IQR 12.1). Both rINSENODIAB and pINSENODIAB cohorts were comparable for gender, BMI SD, presence of DKA, glycemia (mg/dL) at admission, and TIDD_d_ (IU/kg BW/day). Baseline characteristics of both cohorts are summarized in Table 1.

### 3.2. Evolution and Prediction of TIDD during the Initial Hospitalization

**Figure 2:**
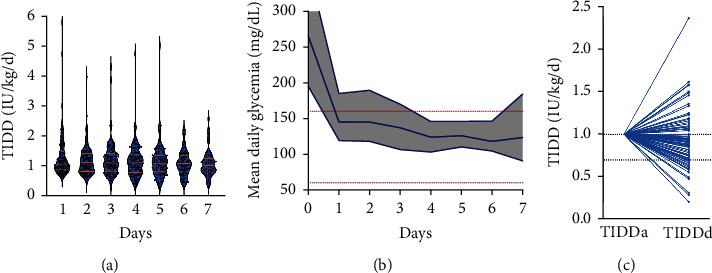
Insulin (a) and glycemic (b) variations throughout the hospitalization and between admission and discharge (c).

On admission, patients with new-onset T1D received an insulin dose based on weight and then adjusted to their blood glucose levels. Since TIDD_d_ reflects improved glycemic control, we used it to elaborate a predictive model of TIDD at admission for new-onset T1D. To determine whether TIDD_d_ is related to TIDD_a_, we first assessed the longitudinal variability of the TIDD and blood glucose throughout the initial hospitalization. We noticed that all patients were euglycemic on the third day of hospitalization with a median glycemia of 137.0 mg/dL (range 106.5-170.0 mg/dL) and that the mean daily glycemia was increasing afterwards for patients hospitalized more than seven days (Figures 2(a) and 2(b)). Median TIDD_a_ was 1.3 IU/kg BW/day while median TIDD_d_ was 1.1 IU/kg BW/day. Using delta TIDD to study the correlation between the two measures, we found that TIDD was slightly but significantly higher upon admission than at discharge (*p* < 0.001) with a median delta TIDD of -0.19 IU/kg BW/day (IQR 0.35) or -2.0 IU/day (95% CI -4.5; -1.0) (Figure 2(c)).

Since there is a significant difference between TIDD_a_ and TIDD_d_, we wanted to further describe TIDD variability throughout the hospitalization using subgroups based on baseline characteristics at diagnosis, including age (5 years, 5-10 years, and >10 years), gender, BMI SD (-2 SD, -2 SD–+1.6 SD, and > +1.6 SD), symptoms duration (2 weeks, 2-4 weeks, 1-2 months, and >2 months), and presence or absence of weight loss or DKA. A multivariable analysis was performed to assess the impact of these characteristics on delta TIDD and, thus, on the risk of variation in the insulin requirement throughout the hospitalization. Interestingly, age, gender, and DKA were found to be important predictors of TIDD variations throughout hospitalization (*p* < 0.05), with the greatest changes reported in children aged 10 and older, male, and with DKA at admission. Results are summarized in Table S1 in the Supplementary Material section.

As patients with DKA were treated with IV insulin upon admission, we also analyzed the effects of IV insulin treatment on TIDD_a_, TIDD_d_, and delta TIDD. The number of days of IV insulin administration during DKA was 1.30 in average (range 1-4). Median TIDD_a_ (IU/kg BW/day) for the IV regimen subgroup was 1.70 (IQR 0.90), as compared to 0.98 (IQR 0.37) for patients under SC insulin regimen (*p* = <0.0001). At discharge, TIDD_d_ (IU/kg BW/day) was still significantly higher in the DKA subgroup with a median TIDD_d_ of 1.25 (IQR 0.57) versus 0.95 (IQR 0.45) in the non-DKA subgroup (*p* = <0.0001).

Although there is a difference in the TIDD between patients with or without DKA, delta TIDD did not significantly differ with the number of days of IV insulin (*p* = 0.20).

As a next step, we developed a predictive model of TIDD based on a combination of clinical and paraclinical factors at admission reflecting the patient's acute clinical state and TIDD_d_ (IU/day), as insulin dose is already adjusted to patient requirements to reach euglycemia. Through a backward stepwise regression on the rINSENODIAB cohort, we found the square of age (*p* < 0.001), percentage of weight loss (*p* < 0.001), veinous pH (*p* = 0.02), veinous bicarbonates (*p* < 0.001), and weight (*p* < 0.001) to be significant predictors of TIDD (IU/day) (Table 2), which should allow to predict the TIDD using the following equation:

(1) $$\begin{array}{c} \text{TIDD}\left(\frac{\text{IU}}{\text{day}}\right)=\left(0.09\times {\text{Age}}^{2}\right)+\left(0.68\times \%\text{Weight Loss}\right)+\left(28.60\times \text{Veinous pH}\right)–\left(1.03\times \text{Veinous bicarbonates}\right)+\left(0.81\times \text{Weight}\right)–194.63. \end{array}$$

Applying the predictive model to the retrospective cohort showed a significant Pearson correlation coefficient of 0.88 (95% CI: 0.83; 0.92) between the predicted and observed values (see Figure S1A in Supplementary Material).

### 3.3. Predictive Model Cross-Validation

To validate the robustness of the model, the pINSENODIAB cohort was investigated as a test set. Of the 80 patients enrolled, 58 had complete data for the relevant parameters in the predictive model. When applying the model equation (Eq. 1) to the test set, we obtained a Pearson correlation coefficient of 0.74 (95% CI: 0.59; 0.84) between the observed and predicted values (see Figure S1B in Supplementary Material).

In the prospective cohort, we evaluated the potential contribution of additional baseline characteristics unavailable in rINSENODIAB. Adding C-peptide and pubertal status did not improve the predictive model performance.

### 3.4. Insulin Dose Requirements at Admission Are Not Correlated with Patient's PR Outcomes at 3 and 12 Months after Diabetes Onset

**Figure 3:**
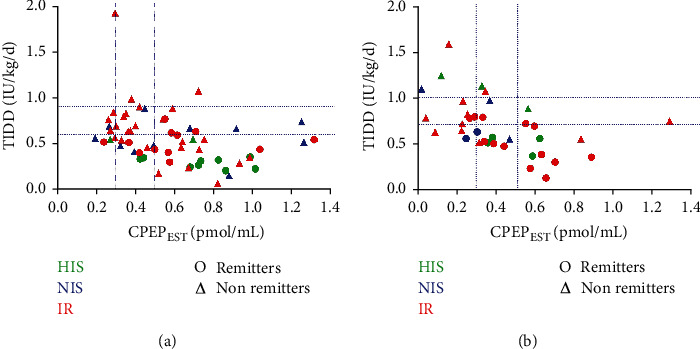
Patient PR outcomes at 3 months (a) and 12 months (b) follow-up.

Partial remission is a transient but beneficial period of T1D likely due to partial recovery of *β* cell function and peripheral insulin resistance, which both partly depend on age or presence of DKA at diagnosis [17]. Out of the 80 patients from the pINSENODIAB cohort, 6 and 34 patients had missing data at months 3 and 12, respectively. To assess the impact of TIDD on patient outcomes at 3 and 12 months after diagnosis, we used HbA_1c_, IDAA_1c_, and CPEP_EST_ levels as biomarkers [30] of PR, with PR defined by an IDAA_1C_ ≤ 9 [30]. Patients were divided into three subgroups depending on their TIDD_d_ (IU/kg BW/day) with (1) highly insulin-sensitive patients (HIS) having a TIDD_d_ below 0.7 IU/kg BW/day (*N* = 13); (2) normosensitive patients (NIS) having a TIDD_d_ in 0.7 to 1.0 IU/kg BW/day range (*N* = 14); (3) insulin-resistant patients (IR) with a TIDD_d_ above 1.0 IU/kg BW/day (*N* = 49). Results are summarized in Figure 3 and Table 3.

At 3 months after diabetes onset, we observed a decrease in insulin TIDD in all sensitivity subgroups, compared to admission. In the IR subgroup, all but three patients (94.12%) achieved a TIDD below 1.0 IU/kg BW/day, with a median TIDD of 0.60 IU/kg BW/day (IQR 0.32). The PR rate was higher in the HIS subgroup (*N* = 10/13; 76.92%) compared to the NIS (*N* = 8/12; 66.66%) and IR subgroups (*N* = 33/51; 64.70%), although not statistically (*p* value 0.71), but with a statistically lower median TIDD in the HIS subgroup (0.37 IU/kg BW/day; *p* = 0.01).

From 3 to 12 months of follow-up, all subgroups shifted towards higher proportions of nonremitters, higher TIDD, and higher HbA_1C_ levels. Although patient's follow-up was limited, we noticed higher IDAA_1C_ (*p* = 0.052) and fewer remitters in the NIS and IR subgroups compared to the HIS subgroups with the higher IDAA_1C_ at month 12 in the NIS subgroup (10.4; IQR 2.9). However, those differences in PR biomarkers were not statistically significant either at 3 or 12 months.

## 4. Discussion

In pediatric patients with new-onset T1D, there are still no consensus guidelines regarding the starting dosing of insulin therapy. The ensuing unpredictability of the initial insulin dose and the following need for dose modifications may contribute to extended hospitalization and increased glycemic variability. In our INSENODIAB study, we first assessed insulin and glycemic variability during hospitalization at T1D onset, showing a slight difference between TIDD_a_ and TIDD_d_. This difference is expected as insulin requirements evolve in parallel to patient's metabolic decompensation and insulin resistance status. Thus, a random assignment of the TIDD upon admission would require daily modifications based on patient clinical parameters, which justifies the search for a more accurate dose calculation algorithm based on patient characteristics. Those characteristics at admission were all considered as dependent variables in the multivariable linear regression used to compute our predictive model. Despite being statistically significant, the difference between TIDD_a_ and TIDD_d_ is clinically minor, allowing us to use TIDD_d_ for our algorithm, as it reflects a daily-basis adjusted insulin dose to reach glycemic control.

According to our study, TIDD could be computed more accurately using the square of age, the percentage of weight loss, the veinous pH and bicarbonates levels, and the weight on admission. The influence of age on daily insulin requirement can probably be explained by the pubertal stage and concentration of contrainsulin-acting hormones [14, 31, 32], responsible for a physiological insulin resistance [33]. Our study showed a univariate effect of the pubertal status on TIDD, and this is in line with the work by Szadkowska et al. [14], who showed that changes in insulin sensitivity in the successive Tanner stages were correlated with changes in insulin dose requirements.

Gender was not found to be a significant predictor of the TIDD in this study, even though age and sex were previously shown to influence insulin resistance and dose requirements, with girls being more insulin resistant than boys [12, 14, 34]. However, an effect of gender on insulin dose requirements is often linked to age and puberty [33]. It is possible that the effect of gender on insulin dose requirements was confounded by age. Furthermore, the difference due to sex is most likely related to visceral deposition and adipose tissue distribution [14]. The fact that fat-free mass was not measured in the current study might account for that discrepancy.

Veinous pH and bicarbonates serum levels are defining markers of the DKA status. In the current study, veinous pH and bicarbonates at admission were found to have a significant and independent correlation with the TIDD. This correlation aligns with our expectations, considering the pathophysiology of T1D and the presence of insulin resistance at onset. Muller et al. showed a strong correlation between insulin dose requirements and ketones concentration, which correlates strongly with veinous bicarbonates and veinous pH [7]. Weitzela et al. also showed that patients with poorer metabolic control at presentation required more insulin [31].

As previously stated, multiple studies suggest that metabolic status parameters have a significant impact on insulin dose requirements [7, 14, 31]. According to our findings, the percentage of weight loss at admission is an important predictor of TIDD. This is not surprising given that it reflects the acute metabolic imbalance due to T1D onset. To our knowledge, this has never been shown in other publications.

The influence of body weight on insulin dose requirements is evident in our study. This fact has been well-established in existing literature, and the guidelines recommend weight-based dosing [13]. Body weight is easily measured and has a strong relationship to both pharmacokinetics and metabolism [35].

Prior studies have documented that intensive insulin therapy can improve endogenous insulin secretion, leading to improved metabolic control [36] and an earlier regulation of blood glucose levels. Interestingly, in our INSENODIAB study, median TIDD_d_ was 1.1 IU/kg BW/day (IQR 0.5) while the major guidelines recommend doses ranging from 0.5 to 1.0 IU/kg BW/day. This is in line with the work of Bag et al. who showed that a higher insulin dose at diabetes onset is associated with better glycemic control in pediatric patients [37]. By using a higher TIDD, we did not provoke an increased rate of hypoglycemia (11.6%) as it was previously described in the literature [31, 38]. This is an important information as several studies have shown that fear of hypoglycemia is the main barrier for tight glycemic control in pediatric patients.

At last, despite variations in both intensity and duration, PR is defined by minimal levels of glycemic variability and insulin requirements. As such, it represents a key period in the early management of diabetes, and early identification of remitters could help develop secondary T1D prevention strategies. Mørk et al. recently studied the impact of insulin sensitivity on the partial remission phase, defined as IDAA_1C_ ≤ 9. They found that nonremitters had significant lower insulin sensitivity up to 14.5 months after diagnosis compared to participants in PR [39]. However, to our knowledge, the impact of TIDD on PR has never been studied. Comparing different insulin-sensitivity subgroups (i.e., HIS, NIS, and IR) in terms of HbA_1c_, IDAA_1c_, and CPEP_EST_ levels at 3 and 12 months after diabetes onset, we showed that the occurrence and duration of PR were not significantly different in all TIDD subgroups. This result suggests that TIDD_d_ mainly reflects an acute metabolic state at diabetes onset. However, we showed a significant difference in TIDD at month 3 after diabetes onset with either a higher TIDD in the IR subgroup or a higher proportion of patients with TIDD > 1 IU/kg BW/d. This could reflect a particular patient phenotype of insulin resistance or disease evolution and will need further attention.

Our analysis of the INSENODIAB cohort demonstrates several strengths. Prediction of TIDD is based on euglycemic patients only and regroups easy-to-collect data only. Its prospective cohort enabled us to cross-validate alternative models and fine-tune our predictive algorithm. Moreover, this is the first pediatric study that assesses TIDD and insulin sensitivity's impact on partial remission. Although this cross-validated study enlightens important predictors of TIDD, further development is recommended to use our predictive model for a correct calculated insulin dose. However, it should be pointed out that patients treated by CSII were not studied separately while being integrated to the predictive model. Also, a further study of patient's phenotypic and genotypic characteristics might help fine-tune our predictive model, as it is recommended by precision medicine.

## 5. Conclusion

In conclusion, in children with new-onset T1D, our study identified key influencing factors for determining optimal TIDD, including the square of age, percentage of weight loss, veinous pH and bicarbonates, and weight. These findings have paved the way for the development of a dosing algorithm that might reduce the time currently needed to stabilize glycemic control and help transition diabetes care to a more individualized approach. Moreover, TIDD_d_ did not influence glucose homeostasis markers at 3 and 12 months after diabetes onset, suggesting that TIDD at diagnosis mainly reflects an acute metabolic state rather than a particular phenotype.

## Figures and Tables

**Table 1:** Demographic, clinical characteristics, and paraclinical measurements.

Characteristics	Global	rINSENODIAB	pINSENODIAB	*p* value^∗^
	(*N* = 183)	(*N* = 103)	(*N* = 80)	
*Distribution*				
Age (years)	9.7 (6.3)	9.2 (6.9)	10.6 (5.6)	0.01^†^
*<5* years*–no. (%)*	*31.0 (17.0)*	*23.0 (22.3)*	*8.0 (10.1)*	
*5-10* years*–no. (%)*	*62.0 (34.0)*	*37.0 (35.9)*	*25.0 (31.6)*	
*>10 years–no. (%)*	*89.0 (48.9)*	*43.0 (41.7)*	*46.0 (58.2)*	
Presence of puberty–no. (%)	NA	NA	38.0 (49.4)	NA
Sex (males) no. (%)	83.0 (45.4)	45.0 (43.7)	38.0 (47.5)	0.6^‡^
Weight (kilograms)	30.0 (21.9)	27.0 (20.9)	32.1 (18.7)	0.03^†^
BMI (*Z*-score)	-0.9 (2.1)	-1.1 (2.1)	-0.7 (1.9)	0.4^†^
*< -2 SD–no. (%)*	38.0 (20.8)	23.0 (22.3)	15.0 (19.0)	
*-2 SD- +1.6 SD–no. (%)*	135.0 (74.2)	74.0 (71.8)	61.0 (77.2)	
*> +1.6 SD–no. (%)*	9.0 (5.0)	6.0 (5.8)	3.0 (3.8)	
*Baseline diabetes characteristics*				
Weight loss (%)	10.0 (10.4)	8.7 (8.6)	12.8 (12.1)	0.007^¶^
HbA_1c_ (%)	11.7 (2.8)	11.5 (2.6)	12.6 (2.7)	0.01^¶^
Presence of DKA–no. (%)	65.0 (35.9)	37.0 (35.9)	28.0 (35.9)	1.0^‡^
Glycemia (mg/dL)	458.5 (241.0)	459.0 (205.0)	457.0 (272.0)	0.8^†^
CPEP_BASAL_ (pmol/mL)	NA		0.2 (0.2)	
*Glycemic data at discharge*				
Insulin total daily dose (IU/kg/day)	1.1 (0.6)	1.1 (0.5)	1.1 (0.6)	0.1^†^
Insulin regimen				
*MDI–no. (%)*	145.0 (79.2)	95.0 (92.2)	49.0 (62.0)	
*Insulin pump–no. (%)*	17.0 (9.3)	8.0 (7.8)	9.0 (11.4)	
*Unknown–no. (%)*	21.0 (11.5)	0.0 (0.0)	21.0 (26.6)	
Normoglycemic patients at discharge (70-180 mg/dL)–no. (%)	NA	94.0 (91.3)		
Hospitalization time (days)	NA	6.0 (5.0)		

Legend: values are median and IQR. Percentages may not total to 100 because of rounding. ^∗^*p* values calculated between subgroup results were considered as significant when under 0.05. ^†^Student *t*-test, ^‡^chi-square, ^¶^Wilcoxon test. HbA_1C_: glycated hemoglobin level; DKA: ketoacidosis; MDI: multiple daily injection; NA: not applicable.

**Table 2:** Stepwise linear regression analysis used to assess determinants of the TIDD (IU/day).

Dependent variable	Coefficient	SE coefficient	*p* value
Intercept	-194, 62	83, 27	0.021801
Age^2^ (years)	0.09020	0.02727	0.001385
Weight loss (%)	0.67817	0.16919	0.000132
Veinous pH	28.59818	11.87413	0.018208
Veinous Bicarbonate (mmol/L)	-1.02781	0.26083	0.000167
Weight (kg)	0.81462	0.13253	2.55*e* − 08

For the whole model: *R*^2^ = 0.7809 and *R*^2^ adjusted = 0.7678; Pearson's correlation coefficient 0.88; *p* value < 2.2*e* − 16.

**Table 3:** ANOVA testing for partial remission outcomes.

*N*	M3				M12			
	76				46			
	HIS	NIS	IR	*p* value^±^	HIS	NIS	IR	*p* value^±^
*N* (%)	13 (17.1)	12 (15.7)	51 (67.1)		9 (19.6)	7 (15.2)	30 (65.2)	
IDAA_1C_	7.2 (1.6)	8.7 (1.6)	8.4 (0.3)	0.052	8.7 (2.4)	10.4 (2.9)	9.2 (1.9)	0.4
*N* remitters (%) (IDAA_1C_ ≤ 9)	10 (76.9)	8 (66.7)	33 (64.7)		5 (55.6)	2 (28.6)	14 (46.7)	
TIDD (IU/kg/d)	0.3 (0.1)	0.6 (0.2)	0.6 (0.3)	0.01^∗^	0.6 (0.4)	0.8 (0.4)	0.6 (0.3)	0.4
TIDD > 1 IU/kg/d	0	0	3 (5.8)		2 (22.2)	2 (28.6)	2 (6.7)	
HbA_1C_ (%)	6.0 (1.1)	6.4 (0.6)	6.2 (0.6)	0.6	7.1 (1.0)	6.5 (1.7)	6.6 (1.3)	0.7
CPEP_EST_ (pmol/mL)	0.7 (0.3)	0.5 (0.5)	0.5 (0.3)	0.2	0.5 (0.2)	0.3 (0.3)	0.3 (0.4)	0.2
N CPEP_EST_ > 0.3 pmol/mL (%)	12 (92.3)	10 (83.3)	34 (66.7)		8 (88.9)	4 (57.1)	20 (66.7)	

Legend: values are median and IQR. Percentages may not total to 100 because of rounding. ^±^One-way ANOVA testing; ^∗^*p* value is considered significant if < 0.05. Abbreviations: IDAA_1C_ = insulin-dose adjusted A_1C_; TIDD = insulin total daily dose; HbA_1C_ = glycated hemoglobin level; CPEP_EST_ = estimated C-peptide; NA = not applicable.

## Data Availability

The data used to support the findings of this study may be released upon application to our PCIC (Pediatric Clinical Investigation Center), whose contact can be reached at the following e-mail address: julia.versavau@uclouvain.be.

## Additional Points

*Key Messages*. In children with new-onset T1D, there is a lack of consensus in the major guidelines (i.e., ISPAD, AACE, ACE, and ADA guidelines) regarding the initial TIDD. Lack of precision of the starting insulin dose may have numerous consequences—all being associated with a delay in achieving metabolic control. According to our study, TIDD could be computed more correctly using the square of age, the percentage of weight loss, the veinous pH, the veinous bicarbonates levels, and the weight at admission. Furthermore, there was no significant link between the initial TIDD and partial remission indicators (IDAA_1C_ and C-peptide) at 3- and 12-months postdiagnosis intervals. This observation could imply that TIDD primarily represents the patient's acute metabolic condition rather than a singular phenotype. These findings helped for the development of a dosing algorithm with the potential to rapidly achieve glycemic control stabilization and facilitate a more individualized approach to treatment regimens.

## Supplementary Materials

Supplementary Materials The supplementary material contains a checklist, one table, and one figure, the first one (Table S1) detailing the influence of age, sex, DKA, BMI SD, and symptoms duration subgroups on the delta total insulin daily dose; the second one illustrates the scatter plots of both the predictive (Figure S1A) and prospective (Figure S1B) model fits.
