# Supplementary material for: Determinants and Characteristics of Insulin Dose Requirements in Children and Adolescents with New-Onset Type 1 Diabetes: Insights from the INSENODIAB Study
Source: J Diabetes Res. 2023 Nov 26;2023:5568663. doi: 10.1155/2023/5568663 (PMC11156506; doi:10.1155/2023/5568663)
Supplement: Supplementary Materials — The supplementary material contains a checklist, one table, and one figure, the first one (Table S1) detailing the influence of age, sex, DKA, BMI SD, and symptoms duration subgroups on the delta total insulin daily dose; the second one illustrates the scatter plots of both the predictive (Figure S1A) and prospective (Figure S1B) model fits. [file 5568663.f1.zip › Table S1.docx]

**Table S1.** **Delta TIDD by subgroups**

| **Dependent variable** | **Delta TIDD mean (CI95%)** | **P value*** |
| --- | --- | --- |
| **Age**  *<5 years old*  *5-10 years old*  *>10 years old* | -1.9 (-6.6;2.7)  -2.3 (-5.4;0.8)  -8.8 (-14.8;-2.8) | 0.04^†^ |
| **Sex**  *Male*  *Female* | -8.8 (-14.2;-3.4)  -2.1 (-5.1;1.0) | 0.02^†^ |
| **DKA**  *Yes*  *No* | -11.5 (-18.3;-4.7)  -1.19 (-3.3;0.9) | 0.0002^†^ |
| **BMI SD**  *< -2 SD*  *-2 - +1.6 SD*  *> +1.6 SD* | NA | 0.4^†^ |
| **Symptoms Duration**  *< 2 weeks*   - 1. *weeks*   2. *months*   *>2 months* | NA | 0.5^†^ |

Legend: values are mean and CI95%. *p value calculated between subgroups results were considered as significant when under 0.05. ^†^ Student t-test. DKA: Ketoacidosis; NA: not applicable.
