# Supplementary figures and images for: Determinants and Characteristics of Insulin Dose Requirements in Children and Adolescents with New-Onset Type 1 Diabetes: Insights from the INSENODIAB Study
Source: J Diabetes Res. 2023 Nov 26;2023:5568663. doi: 10.1155/2023/5568663 (PMC11156506; doi:10.1155/2023/5568663)

**Figure S1. Scatter plot of predictive model (1A) and of prospective model fit (1B)**


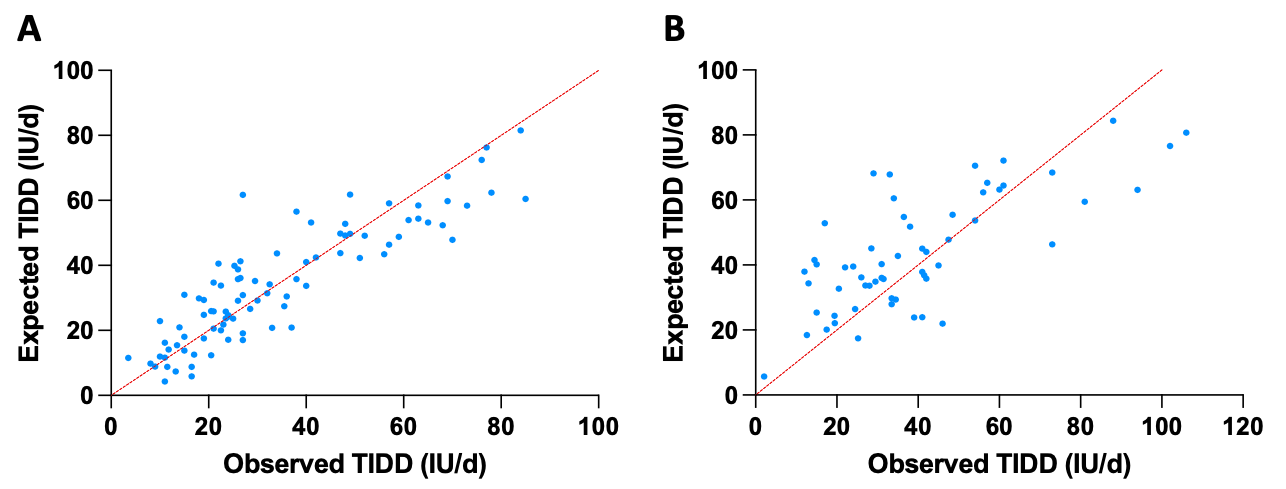

Supplement: Supplementary Materials — The supplementary material contains a checklist, one table, and one figure, the first one (Table S1) detailing the influence of age, sex, DKA, BMI SD, and symptoms duration subgroups on the delta total insulin daily dose; the second one illustrates the scatter plots of both the predictive (Figure S1A) and prospective (Figure S1B) model fits. [file 5568663.f1.zip › Figure S1..docx]
